# The difference in knowledge and concerns between healthcare professionals and patients about genetic-related issues: A questionnaire-based study

**DOI:** 10.1371/journal.pone.0235001

**Published:** 2020-06-19

**Authors:** Basima A. Almomani, Nour A. Al-Sawalha, Maha S. Al-Keilani, Hatem A. Aman

**Affiliations:** 1 Department of Clinical Pharmacy, Faculty of Pharmacy, Jordan University of Science and Technology, Irbid, Jordan; 2 Department of Applied Biological Sciences, Jordan University of Science and Technology, Irbid, Jordan; Universiteit Maastricht, NETHERLANDS

## Abstract

Effective adoption of genetics in clinical practice requires the support of and interaction between the different partners of healthcare system; healthcare professionals (HCPs) and patients. The study aimed to assess and compare the knowledge, factors affecting the knowledge, and concerns of HCPs and patients regarding genetic-related issues such as lack of knowledge about genetics and genetic conditions, awareness of the importance of genetics in clinical practice and genetic services and resources deficits. A cross sectional study was conducted in different areas of Jordan using a convenient sampling approach. An English questionnaire was self-administered to HCPs. Face-to-face interviews were conducted with patients in Arabic by trained researcher. A total of 1000 HCPs and 1448 patients were recruited. There was a significant difference (p<0.001) in the knowledge between HCPs and patients. Among HCPs, physicians (OR = 2.278, 95%CI = 1.410–3.680, p = 0.001) and pharmacists (OR = 2.163, 95%CI = 1.362–3.436, p = 0.001) were more knowledgeable than nurses. In addition, females were more knowledgeable than males (OR = 1.717, 95%CI = 1.203–2.451, p = 0.003). Among patients, participants who had a bachelor degree (OR = 1.579, 95%CI = 1.231–2.025, p<0.001) were more knowledgeable compared to those who only had school education. HCPs appeared to have more concerns than patients (p<0.001) regarding all genetic-related issues. These findings suggested a positive association between education and genetic knowledge as well as concerns; as HCPs were more knowledgeable and concerned than patients. Appropriate integration and expansion of basic genetic knowledge courses and clinical genetic training in the curriculum should be adopted to prepare HCPs to enhance the integration of genetic information in clinical settings.

## Introduction

New scientific insights about genetics generate contemporary medical opinion, which influences the decision-making process of patients and healthcare professionals (HCPs). The majority of the studies that aimed to build-up the genetic inventory of human genome and understand the ethnicity-disease relationship were implemented in the developed countries where the current genomic database was built using the western population. Therefore, there is an urgent need to conduct genetic studies in the Middle Eastern countries to create a catalog of Eastern genome data. Several studies were performed in the Middle Eastern countries to build the genome database of the Arab populations [1–4], but sufficient data are not yet available to capture the genetic diversity for Arabs. Accordingly, for this to be achieved and for the successful adoption of genetic researches, HCPs and people should have the sufficient knowledge and awareness about genetics and its new technologies.

It was reported that a higher level of knowledge was linked to positive attitudes toward genetic testing [5]. The majority of HCPs in New York City, many of whom were junior physicians, received genetic education and they showed positive views toward testing patients for genetic susceptibility to develop chronic diseases [6]. Further, Puryear and colleagues showed that patients and HCPs expressed their concerns about the impact of genetic differentiation on social inequality and stereotyping in many situations such as health and life insurance as well as employment opportunities [7]. Fears from genetic discrimination as well as its effect on insurance coverage and employment chances were raised by different countries and led to the adoption of different policies to ensure the fairness and usefulness of the genetic information [8].

Horowitz and colleagues showed that patients who were at genetic risk of developing renal failure believed that utilization of genetic information could motivate HCPs to provide a better care [9]. With advances in medical genetics, HCPs will be involved in delivering personalized medicine through advising patients and interpreting the test’s results [10]. Patients with chronic diseases rely primarily on HCPs to control their conditions and hence their knowledge could be affected by HCPs. However, patients depend on other resources such as internet and social media among others [11–13]. Delikurt and colleagues reported that the lack of genetic knowledge among both patients and HCPs was a barrier to prevent the patients from referral to genetic services in the United stated and European countries [14]. Therefore, it is necessary that HCPs are equipped with appropriate genetic knowledge to apply complex information and take into consideration the values and needs of each patient [15]. Despite the fact that the data regarding the knowledge of and attitudes towards genetics among HCP and patients is available in the literature [7, 15–19], the implementation of genetic services using DNA-technologies is different among countries. This could be affected by the availability and accessibility of these services, national polices, in addition to the cultural and religious issues. In Jordan, genetic application is still at the beginning and more genetic research is needed. The current study aimed to assess and compare the knowledge, factors affecting knowledge and concerns of both HCPs and patients regarding issues related to the application of genetics information in clinical settings. The results of the current study could assist the decision makers to adopt educational training programs to enhance the acceptance of genetic concepts that would further enhance the acceptability of applying genetics in the clinical practice.

## Materials and methods

### Ethics statement

The ethical approval to conduct this research was granted by the institutional review boards in Jordan University of Science and Technology (156/2017) and the Ministry of Health (10411). The informed consent was implied as the participant agreed to participate in the study and the data were analyzed anonymously.

### Study design and subjects

A cross sectional study was conducted in different areas in Jordan using a convenience sampling approach. The participants (HCPs and patients with chronic disease) were recruited from different hospitals in Jordan (n = 7). Two sampling methods were employed for data collection. An English questionnaire was self-administered to HCPs in which the questionnaire was completed by the participants without the intervention of the trained researcher (a Pharm D holder). The face-to-face interviews were conducted with patients in Arabic by trained researcher to ensure consistent method of data collection and avoid any missing information in answered questionnaire. A total of 1700 patients and 1300 HCPs were approached. Of these, 1448 patients agreed to participate with a response rate of 85.2% while 1000 HCPs returned the filled questionnaire with a response rate of 76.9%.

### Questionnaire development

The questionnaire was developed after reviewing the literature for related topics. Some items of the questionnaire were selected from a previous study [19] and other items were newly developed after a thorough discussion within the research team. The questionnaire consisted of three sections that addressed (i) knowledge (ii) concerns, and (iii) the demographics. Both face and content validity were assessed by two experts in the field. The items of questionnaire were revised based on their opinion and comments. The questionnaire was originally written in English for HCPs (S1 File) and then translated to Arabic for patients (S2 File). Both forward (English to Arabic) and backward (Arabic to English) translation were carried out by two independent researchers who reported a high match percentage between the two drafts.

The questionnaire was piloted to a number of participants (n = 15 per each) to identify any unclear questions or potential problems with the questions that might cause biased answers. The adjustments and modifications were made to enhance the clarity of the questionnaire. The data from piloting was not included in the final analysis. About 10 minutes were needed to complete the questionnaire. The informed consent was taken as implied via returning the filled questionnaire by HCP and giving oral approval by patient.

All items in the questionnaire were formatted as close-ended questions. The knowledge section was composed of 7 items that had three possible answers (correct, incorrect, I do not know). The concern section was composed of 7 statements with a 5-point Likert scale (ranging from "very unconcerned" to "very concerned"). The internal consistency using the Cronbach’s α (alpha) measure of related sections in the questionnaire was 0.650 which indicated good reliability.

### Statistical analysis

All analyses were conducted using Statistical Package for Social Sciences (SPSS Inc., Chicago, IL) version 20. Counts and percentages were used for categorical variables and median [Interquartile range] was used for continuous variables. Shapiro–Wilk tests was used to test for normality of continuous variables. Chi-square test was used for categorical variables and Mann–Whitney U test was used for non-parametric continuous variables in the univariate analysis. After that, multivariate analysis using binary logistic regression (LR) was performed to determine factors that were independently associated with the participants' knowledge and to calculate odds ratio (OR) and 95% confidence intervals (95% CI). All variables with p<0.25 on univariate analysis were included in the multivariate analysis. Statistical significance was set at p <0.05.

To include knowledge in the binary LR model, it was computed into a binary variable: knowledgeable if the sum of the scores was ≥ 5 (out of 7) and non-knowledgeable if the sum of the scores was < 5 (out of 7). The responses to the 7 statements of knowledge for each participant were categorized using a cut-off point for the total scores of correct answers. All participants who stated “do not know” were coded as “incorrect”. For the purpose of analysis, the response options for the concern section were restricted as both "very unconcerned" and "unconcerned" were combined as one category and both "very concerned" and "concerned" as another category.

## Results

### Demographics

A total of 1000 and 1448 questionnaires were collected from both HCPs and patients respectively. The median age of HCPs was 30 years and more than half were females. HCPs were categorized as nurses (38.8%), physicians (33.9%) and pharmacists (27.3%) with the average of 5 years of experience. The median age of patients was 49 years with 60% of them were females. Three quarters of patients had one chronic disease; cardiovascular diseases (46.3%) and diabetes mellitus (46.8%) were the two most common conditions. One third of patients had a bachelor's degree (32.3%), three quarters were married (73.8%), and about 70% of patients had a monthly income of average less than 500 Jordanian Dinar (JD). Detailed demographics for both HCPs and patients are presented in Table 1.

**Table 1:** Demographic of the participants.

Characteristics ^a^	
**Healthcare professionals n = 1000**	
Age ^b^	30 [26–36]
Gender	
•Male	398 (39.8)
•Female	602 (60.2)
Family monthly income	
•<500 JD	350 (35)
•500–1000 JD	423 (42.3)
•>1000 JD	227 (22.7)
HCP's category	
•Nurse	388 (38.8)
•Pharmacist	273 (27.3)
•Physician	339 (33.9)
Years of experience ^b^	5 [2–12]
**Patients n = 1448**	
Age ^b^	49 [39–58]
Gender	
•Male	562 (38.8)
•Female	886 (61.2)
Type of chronic disease	
•Cardiovascular disease	671 (46.3)
•Diabetes mellitus (type 1 & 2)	677 (46.8)
•Respiratory	240 (16.6)
•Others	323 (22.3)
Number of chronic conditions	
•One condition	1077 (74.4)
•≥ two conditions	371 (25.6)
Level of education	
•School education	981 (67.7)
•Bachelor's degree	467 (32.3)
Marital Status	
•Single	223 (15.4)
•Married	1069 (73.8)
•Other (widowed/ divorced)	156 (10.8)
Family monthly income	
•<500 JD	1005 (69.4)
•500–1000 JD	395 (27.3)
•>1000 JD	48 (3.3)

HCP, Healthcare professional; JD, Jordanian Dinar

^a^ All data were expressed as n (%) of participants unless otherwise indicated

^b^ Data were described as median [*Interquartile* range]

### Genetic knowledge

**Fig 1:**
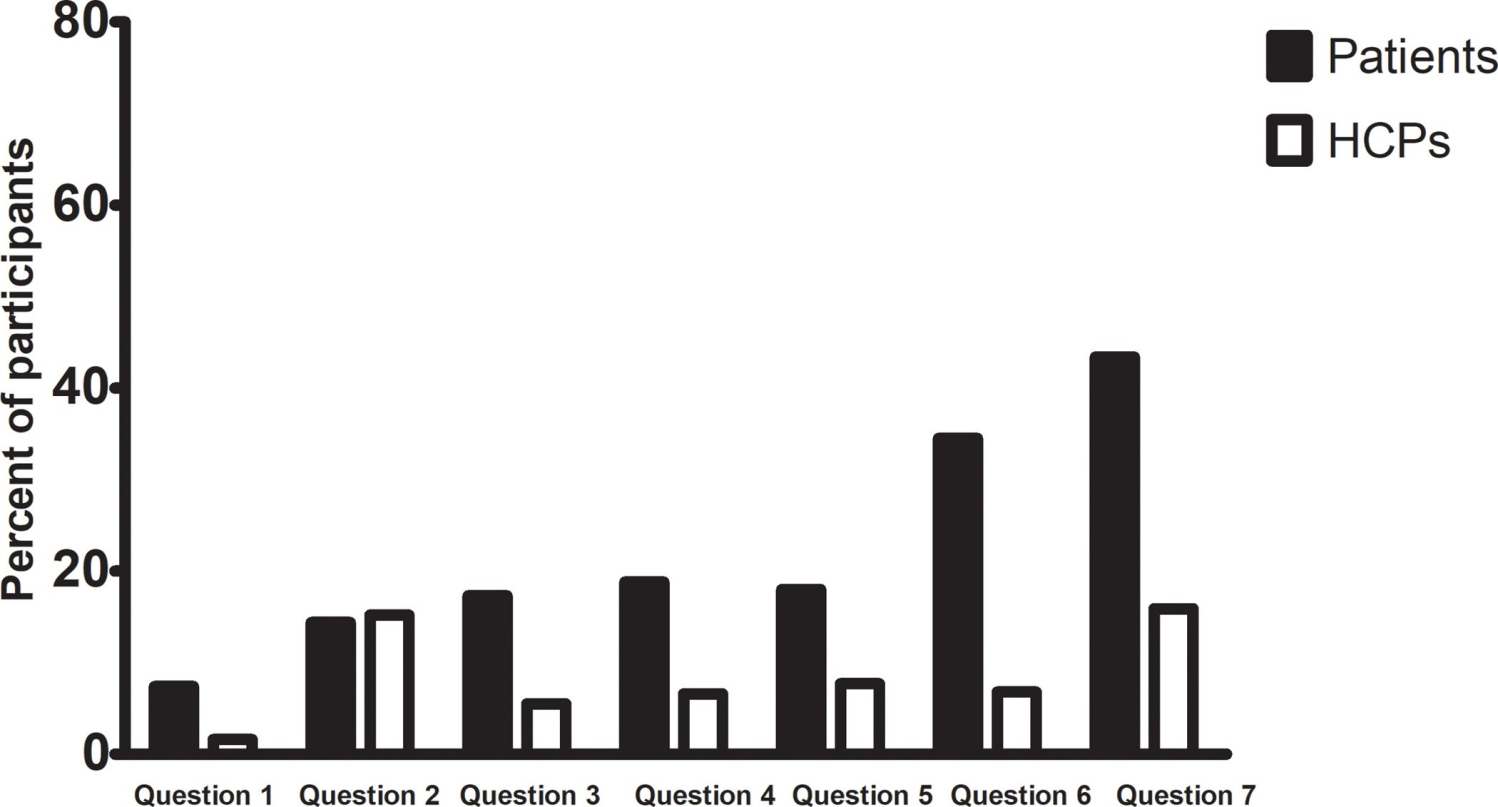
Percentages of participants who answered "do not know" for knowledge questions.

The majority of HCPs (81.7%, n = 817) were knowledgeable and the mean number of correct answers was 5.56 (range from 0 to 7). A quarter of HCPs (26.6%, n = 266) answered all questions correctly and few participants (0.3%, n = 3) did not respond correctly to any question (knowledge score = 0). On the other hand, less than half of patients (41.4%, n = 599) were knowledgeable with a mean number of correct answers of 4.02 (range from 0 to 7). Only 5.3% (n = 77) of patients answered all questions correctly and 1.9% (n = 27) did not respond correctly to any question (knowledge score = 0). There was a significant difference in the number of knowledgeable participants between HCPs and patients (p<0.001). An overview of the questionnaire items and answers is presented in Table 2. There were significant differences in the correct responses between HCPs and patients regarding all knowledge questions (p <0.001) except question 2 (p = 0.190). Fig 1 shows the percentages of participants who answered "do not know" for knowledge questions. Interestingly, a sixth of both study groups were undecided regarding question 2. Knowledge question one "there is a relation between consanguinity and genetic disease" was the one that was most often answered correctly by both groups [95.6% (n = 956) for HCPs and 79.8% (n = 1156) for patients]. On the other hand, question 7 "genetic information will help in predicting drug response to some therapies" had the lowest number of correct answers among patients (27%) and question 2 "patients have the right to refuse genetic-testing" had the lowest number of correct answers among HCPs (48.1%). More than half of HCPs (61.9%) reported gaining their knowledge through medical course/training sessions rather than media and internet (26.7%). On the other hand, almost a third of the patients stated that they gained their knowledge through media (38.1%), other people (21.1%), HCPs (19.1%), or the newspapers (5.1%).

**Table 2:** Overview of knowledge questions for the participants.

Items in the questionnaire	Correct responses For HCPs N (%)	Correct responses For patients N (%)	P-value
1. There is a relation between consanguinity and genetic disease (True)	956 (95.6)	1156 (79.8)	<0.001
2. Patients have the right to refuse genetic-testing (True)	481 (48.1)	736 (50.8)	0.190
3. Healthy parents can have a child with a hereditary disease (True)	883 (88.3)	1028 (71)	<0.001
4. The carrier of a disease gene may be completely healthy (True)	833 (83.3)	1000 (69.1)	<0.001
5. All serious diseases are hereditary (False)	764 (76.4)	756 (52.2)	<0.001
6. Genetic information will help in predicting susceptibility of some disease (True)	870 (87)	757 (52.3)	<0.001
7. Genetic information will help in predicting drug response to some therapies (True)	768 (76.8)	391 (27)	<0.001

The results of univariate analysis showed that age (p = 0.007), gender (p = 0.004), income (p = 0.002), HCPs category (p<0.001) and years of experience (p = 0.002) were significantly associated with the knowledge level of HCPs. However, the results of multivariate analysis identified that gender and HCPs category were the only independent factors affecting the knowledge (Table 3). Females were more knowledgeable than males (OR = 1.717, 95%CI = 1.203–2.451, p = 0.003) and physicians (OR = 2.278, 95%CI = 1.410–3.680, p = 0.001) and pharmacists (OR = 2.163, 95%CI = 1.362–3.436, p = 0.001) were more knowledgeable than nurses. On the other hand, the results of univariate analysis among patients showed that gender (p = 0.043), educational level (p<0.001) and income (p = 0.034) were significantly associated with patients' knowledge. Multivariate analysis indicated that only gender and educational level were independently associated with the patients' knowledge (Table 4). Participants who had a bachelor’s degree were more knowledgeable compared to those who only had school education (OR = 1.579, 95%CI = 1.231–2.025, p<0.001). Females were slightly more knowledgeable than males (OR = 1.378, 95%CI = 1.095–1.733, p = 0.006), however, this finding cannot be translated to practice as the difference is very small.

**Table 3:** Multivariate analysis of factors affecting HCPs’ knowledge.

Factors	OR (95% CI)	P-value
Age	0.954 (0.901–1.010)	0.104
Gender		0.003
•Male	Ref	
•Female	1.717 (1.203–2.451)	
Family monthly income		
•<500 JD	Ref	0.093
•500–1000 JD	1.005 (0.680–1.487)	0.978
•>1000 JD	1.755 (0.987–3.121)	0.056
HCP's category		
•Nurse	Ref	<0.001
•Pharmacist	2.163 (1.362–3.436)	0.001
•Physician	2.278 (1.410–3.680)	0.001
Years of experience^*b*^	1.031 (0.969–1.097	0.329

CI, confidence intervals; HCP, Healthcare professional; JD, Jordanian Dinar OR, odds ratio;

**Table 4:** Multivariate analysis of factors affecting patients’ knowledge.

Factors	OR (95% CI)	P value
Age	0.998 (0.989–1.007)	0.731
Gender		0.006
•Male	Ref	
•Female	1.378 (1.095–1.733)	
Number of chronic conditions		0.109
•One condition	Ref	
•≥ two conditions	1.234 (0.955–1.594)	
Education		<0.001
•School education	Ref	
•Bachelor’s degree	1.579 (1.231–2.025)	
Marital status		
•Single	Ref	0.117
•Married	1.034 (0.741–1.443)	0.844
•Other	0.701 (0.431–1.139)	0.151
Family monthly income		
•<500 JD	Ref	0.213
•500–1000 JD	1.22 (0.955–1.562)	0.112
•>1000 JD	0.859 (0.464–1.590)	0.628

CI, confidence intervals; JD, Jordanian Dinar; OR, odds ratio

### Potential concerns

**Fig 2:**
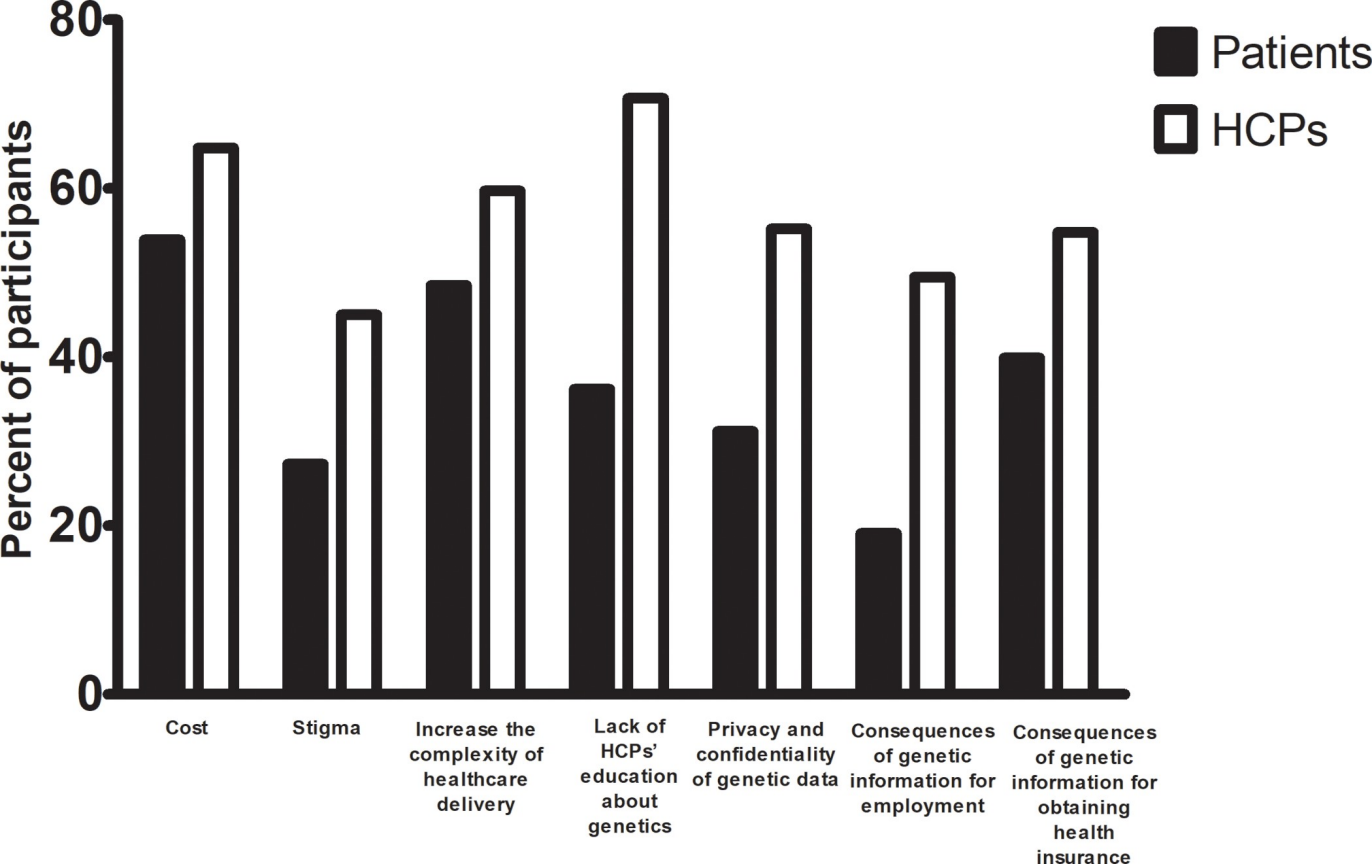
Potential concerns of HCPs and patients to the practical application of genetics in clinical settings.

An overview of participants' responses regarding potential concerns to the practical application of genetics in clinical practice is presented S1 Table. There were variations in the responses between HCPs and patients regarding all genetic-related issues. In general, HCPs appeared to have more concerns than patients (p<0.001). Almost three quarters of the HCPs were concerned that lack of HCP education would be a barrier to the genetic service implementation compared to only one third of the patients (p<0.001) (Fig 2). Furthermore, about half of the HCPs were more concerned about the privacy and confidentiality of genetic information and their related effects on health insurance and employment compared to smaller percentages among the patients (p<0.001) (Fig 2). Approximately, a sixth of patients and a quarter of HCPs answered neutral for all questions as shown in S1 Table.

## Discussion

The current study aimed to assess and compare the knowledge and concerns of HCPs and patients regarding genetic-related issues and it showed three main findings. First, HCPs were more knowledgeable about genetic information and at the same time more concerned about the application of genetics in clinical practice compared to patients. Second, Physicians and pharmacists were more knowledgeable than nurses. Third, patients who had a bachelor's degree were more knowledgeable compared to those who only had school education.

Views and knowledge of patients and HCPs about genetic information and services have been examined in different European countries. [6, 7, 14, 19, 20]. Few studies examined these issues especially in the Eastern Mediterranean region [16, 17]. Despite the fact that genetic knowledge is increasing, this was not the case for patients with chronic diseases who had little knowledge about genetics, as 60% of them were not knowledgeable in the current study. A previous study by Kaphingst and colleagues found that low level of genetic knowledge among patients was associated with limited health literacy [21]. Galesbeek and colleagues also reported the association between genetic knowledge and high level of education [19] which is in line with the findings of the current study. Importantly, the findings of the current study showed a specific gap in genetic knowledge. For example, only 27% of patients and 76% of HCPs reported that genetic information would help in predicting drug response to some therapies. Previous studies highlighted the knowledge gap of HCPs about pharmacogenomic testing and how they would alter the treatment approach [7, 22]. Therefore, more intensive educational programs are needed to support the integration of pharmacogenomics in clinical practice.

Previous studies reported limited genetic knowledge for HCPs [20, 23, 24]. The current study found a high knowledge score among HCPs (5.56 out 7) which might be affected by the nature of the current study, a short questionnaire-based study, as well as the type of the participants as more than half of participants were females. This is supported by a previous study in Saudi Arabia which found that female college students had higher knowledge [25]. In contrast, other reports showed a knowledge gap and lack of a clear guideline for primary care physicians about genetics [6, 22, 26]. The current study showed that nurses were less knowledgeable than physicians and pharmacists, consistent with a previous study in Jordan that showed nurses and midwives had inadequate level of knowledge about genetic teaching [16]. This could be explained by the shortage of genetics content in their curricula and the limited ability of nursing faculty to translate this science to clinical courses [27]. This highlights the need for comprehensive review of the nursing educational program to address this issue.

There are several resources that assist the patients to gain information about genetics [11, 13]. Less than a quarter of the patients in the current study reported that they gained their knowledge through HCPs. A consistent finding by Alnaif and Alghanim who showed that about 20% of patients received health education in primary health-care centers in Saudi Arabia [28]. In addition, Eum and colleagues reported that patients heard about genetics from clinician (38%) and internet (37%) [12]. It was reported that participants with higher genetic knowledge were more likely to select genetic testing for health conditions [29]. However, HCPs reported that they lacked the confidence in translating the genetic information into clinical practice [6, 7]. In the current study, half of both study groups admitted on the patient’s rights to refuse the genetic test. Importantly, pre-test genetic counseling is important to help patient make informed decision to accept or decline testing [10]. The HCPs should take in consideration the patients’ preference regarding genetic counseling [30] and should, at the same time, understand the patients’ feelings upon receiving the results of the genetic test [31]. A recent study in the USA reported that patients were confident in HCPs’ knowledge about genetics and would like to discuss these issues with them [7]. Ongoing training and education is required for HCPs to adapt with the rapidly changing and developing genetic applications [32].

The current study identified concerns of patients and HCPs regarding the practical application of genetics in the medical field. Most of these concerns were raised by both patients and HCPs in a recent study in the USA [7]. In general, HCPs appeared more concerned than patients regarding all genetic-related issues. This is consistent with a recent study in South Korea where the HCPs were more concerned than patients toward genetic testing [12]. The demonstrated high knowledge of HCPs in the current study has the potential to affect their concerns as they may be more realistic about the current situation of genetic application. Additionally, the current study showed that some patients were undecided regarding questions of knowledge (answered "I do not know") and concerns (answered "neutral") and might be unable to comment on this issue as they found it difficult to formulate their opinion. It was reported that 80% of surveyed patients believed in favorable use of genetics in diagnosis, prevention and treatment [7]. Issues of the cost of genetic testing, data privacy and discrimination on insurance coverage and employment were highlighted in previous studies [6, 7, 12, 19]. The cost of genetic testing is a barrier leading to unequal access among different patients, however, the cost is decreasing over time and insurance companies are establishing criteria for cost-management [7]. Interestingly, despite that most of HCPs in the current study were knowledgeable about genetics, they were concerned about the lack of education. Previous study reported that HCPs expressed that they had limited training and they would enhance their knowledge and clinical utility of genetic testing by appropriate genetic training [7]. Clinical utility can be defined as the effectiveness, in terms of risks and benefits, of genetic approach when used in practice compared to the routine care [10]. Time constraints and resources availability were also among the other concerns that were mentioned in the literature [26], consistent with the current study findings as the participants reported that genetic application would increase the complexity of providing healthcare services.

The current study has some limitations. First, the convenience sampling approach that was used in the current study raised the potential of selection bias as the participants who were interested in genetics could have participated more than others. However, our sample included a diversity of HCPs and respondents were recruited from different geographical areas of Jordan (North, South, Middle) with a high response rate among both study groups. In addition, the level of genetic knowledge among patients (41.4%) in the current study was close to the level of genetic knowledge among public (43.4%) in our previous published study in Jordan. Second, the questionnaire was built using questions from another study due to the lack of a validated questionnaire in this topic. However, face and content validity were assessed by the research team in addition to piloting of the questionnaire. Third, dichotomizing people into "knowledgeable" and "non-knowledgeable" has the potential to lose a lot of nuance in the response. However, this was performed to simplify the analysis as 7 questions in knowledge section were examined, besides this is an overview study to assess the basic knowledge rather than focusing on the advanced genetic knowledge of HCPs. Additionally, different studies which investigated the level of knowledge categorized the participants into two groups as well [33, 34]. Fourth, same questions were used for both HCPs and patients, however, previous study by Eum and colleagues used similar type of questions for both HCPs and patients [12].

## Conclusions

This is the first study comparing knowledge and concerns of patients and HCPs toward genetics related issues. Compared to patients, HCPs were more knowledgeable and more concerned about the application of genetics in clinical practice. In addition, female gender was a predictor for genetic knowledge among HCPs. This study encourages the collaboration and interaction of different partners of genetic services such as HCPs and patients to support the potential utilization and application of genetics in clinical practice. Future studies are recommended to assess the views of HCPs about the effectiveness of genetic approach and if they are ready to adopt it into routine clinical practice. This provides a helpful evidence for policy making regarding how the process of patient care will be altered and affected the clinical decision making.

## Supporting information

S1 File English questionnaire for healthcare professionals. (DOCX)

S2 File Arabic questionnaire for patients. (DOCX)

S1 Table Potential concerns of HCPs and patients to the practical application of genetics in clinical settings. (DOCX)
