# Supplementary material for: The difference in knowledge and concerns between healthcare professionals and patients about genetic-related issues: A questionnaire-based study
Source: PLoS One. 2020 Jun 19;15(6):e0235001. doi: 10.1371/journal.pone.0235001 (PMC7304621; doi:10.1371/journal.pone.0235001)
Supplement: S1 File — (DOCX) [file pone.0235001.s001.docx]

**Genetic-related issues: Knowledge and concerns of Healthcare professionals**

**Demographics**

| HCP study code |  |
| --- | --- |
| - Age |  |
| - Gender | 1. Female 2. Male |
| - Family monthly Income | 1. less than 500 JD 2. 500-1000 JD 3. more than 1000 JD |
| HCP category | 1. Physician 2. Pharmacist 3. Nurse |
| - Years of experience |  |
| Source of information about genetics | 1. Medical courses/ training sessions  2. Media and internet: TV, radio, twitter, facebook, whatsup.  3. Other sources |

**Knowledge about genetic information**

Do you know that …

|  | **0.incorrect** | **1.correct** | **2. I don’t know** |
| --- | --- | --- | --- |
| Q1. There is a relation between consanguinity and genetic disease |  |  |  |
| Q2. Patients have the right to refuse genetic-testing |  |  |  |
| Q3. Healthy parents can have a child with a hereditary disease |  |  |  |
| Q4. The carrier of a disease gene may be completely healthy |  |  |  |
| Q5. All serious diseases are hereditary |  |  |  |
| Q6. Genetic information will help in predicting susceptibility of some disease |  |  |  |
| Q7. Genetic information will help in predicting drug response to some therapies |  |  |  |

**Concerns about genetic-related issues**

To apply genetic approach in clinical settings, please indicate your concern level for the following statements:

|  | **0.very non concerned** | **1.non concerned** | **2.Neutral** | **3.concerned** | **4.very concerned** |
| --- | --- | --- | --- | --- | --- |
| 1.Cost of genetic-testing |  |  |  |  |  |
| 2.Stigmatization |  |  |  |  |  |
| 3.Increasing the complexity of healthcare delivery |  |  |  |  |  |
| 4.Lack of healthcare professionals’ education about genetics |  |  |  |  |  |
| 5.Privacy and confidentiality of genetic data |  |  |  |  |  |
| 6.Consequences of genetic-testing for employment |  |  |  |  |  |
| 7.Consequences of genetic-testing for obtaining health insurance |  |  |  |  |  |
