# Supplementary material for: The difference in knowledge and concerns between healthcare professionals and patients about genetic-related issues: A questionnaire-based study
Source: PLoS One. 2020 Jun 19;15(6):e0235001. doi: 10.1371/journal.pone.0235001 (PMC7304621; doi:10.1371/journal.pone.0235001)
Supplement: S2 File — (DOCX) [file pone.0235001.s002.docx]

**القضايا المتعلقة بعلم الوراثة: المعرفة ومخاوف المرضى الذين يعانون من أمراض مزمنة**

**المعلومات السكانية**

| رمز المشارك |  |
| --- | --- |
| العمر |  |
| الجنس | 1. أنثى 2. ذكر |
| مستوى التعليم | 1. تعليم مدرسي 2. تعليم جامعي |
| الدخل الشهري | 1. اقل من 500 دينار 2. 500-1000 دينار 3. اكثر من 1000 دينار |
| الحالة الاجتماعية | 1. أعزب 2. متزوج 3. أخرى.............. |
| نوع المرض المزمن | 1-السكري  2-امراض تنفسية  3-امراض قلب  4-اخرى ............................. |
| مصدر معلوماتك عن المعلومات الجينية | 1. وسائل الإعلام والتواصل الإجتماعي و مواقع تصفح الإنترنت (التلفاز، الراديو، تويتر، فيس بوك، واتس......) 2. الجرائد 3. الناس 4. مقدمي الرعاية الصحية 5. مصادر أخرى |

**المعرفة حول علم الوراثة**

**هل تعرف ان ...**

|  | **0-خطأ** | **1-صح** | **2-لا أعرف** |
| --- | --- | --- | --- |
| س1. هناك علاقة بين زواج القرابة والأمراض الوراثية |  |  |  |
| س2. لديك الحق في رفض إجراء فحص جيني وراثي لك |  |  |  |
| س3. يمكن للوالدين الصحيين أن يكون لديهم طفل مصاب بمرض وراثي |  |  |  |
| س4. قد يكون الإنسان الحامل لجين مرضي، صحي تماما |  |  |  |
| س5. جميع الأمراض الخطيرة وراثية |  |  |  |
| س6. يمكن التنبؤ بحصول بعض الامراض عن طريق المعلومات الوراثية |  |  |  |
| س7. يمكن التنبؤ باستجابة المريض للدواء عن طريق المعلومات الوراثية |  |  |  |

**مخاوف بشأن علم الوراثة**

يرجى تحديد مدى قلقك تجاه ما يلي في حال تطبيق علم الوراثة في المجالات السريرية

|  | **0-غير قلق على الاطلاق** | **1-غير قلق** | **2-محايد** | **3-قلق** | **4-قلق جدا** |
| --- | --- | --- | --- | --- | --- |
| س1. تكلفة الفحص الجيني الوراثي |  |  |  |  |  |
| س2. الخوف من نظرة المجتمع اذا كنت حامل لجين المرض/ وصمة عار |  |  |  |  |  |
| س3. زيادة الاجراءات اللازمة لتقديم الرعاية الصحية |  |  |  |  |  |
| س4. عدم وجود تعليم لمتخصصي الرعاية الصحية حول علم الوراثة |  |  |  |  |  |
| س5. خصوصية وسرية البيانات الوراثية |  |  |  |  |  |
| س6. عواقب الاختبارات الجينية على التوظيف |  |  |  |  |  |
| س7. عواقب الاختبارات الجینیة للحصول على التأمین الصحي |  |  |  |  |  |
