# Supplementary material for: The difference in knowledge and concerns between healthcare professionals and patients about genetic-related issues: A questionnaire-based study
Source: PLoS One. 2020 Jun 19;15(6):e0235001. doi: 10.1371/journal.pone.0235001 (PMC7304621; doi:10.1371/journal.pone.0235001)
Supplement: S1 Table — (DOCX) [file pone.0235001.s003.docx]

S1 Table. Potential concerns of HCPs and patients to the practical application of genetics in clinical settings

| **P value** | **HCPs n=1000**  **N (%)** | **Patients n=1448**  **N (%)** | **Concerns' level** | **Concerns** |
| --- | --- | --- | --- | --- |
| p<0.001 | 141 (14.1) | 437 (30.2) | Very unconcerned/unconcerned | Cost |
|  | 211 (21.1) | 230 (15.9) | Neutral |  |
|  | 648 (64.8) | 781 (53.9) | Very concerned/concerned |  |
| p<0.001 | 344 (34.4) | 844 (58.3) | Very unconcerned/unconcerned | Stigma |
|  | 206 (20.6) | 208 (14.4) | Neutral |  |
|  | 450 (45.0) | 396 (27.3) | Very concerned/concerned |  |
| p<0.001 | 162 (16.2) | 502 (34.7) | Very unconcerned/unconcerned | Increase the complexity of providing healthcare |
|  | 241 (24.1) | 243 (16.8) | Neutral |  |
|  | 597 (59.7) | 703 (48.5) | Very concerned/concerned |  |
| p<0.001 | 137 (13.7) | 636 (43.9) | Very unconcerned/unconcerned | Lack of HCPs' education about genetics |
|  | 156 (15.6) | 288 (19.9) | Neutral |  |
|  | 707 (70.7) | 524 (36.2) | Very concerned/concerned |  |
| p<0.001 | 269 (26.9) | 840 (58) | Very unconcerned/unconcerned | Privacy & confidentiality of genetic data |
|  | 179 (17.9) | 156 (10.8) | Neutral |  |
|  | 552 (55.2) | 452 (31.2) | Very concerned/concerned |  |
| p<0.001 | 241 (24.1) | 949 (65.5) | Very unconcerned/unconcerned | Consequences of genetic information for employment |
|  | 264 (26.4) | 222 (15.3) | Neutral |  |
|  | 495 (49.5) | 277 (19.1) | Very concerned/concerned |  |
| p<0.001 | 220 (22) | 671 (46.3) | Very unconcerned/unconcerned | Consequences of genetic information for obtaining health insurance |
|  | 232 (23.2) | 199 (13.7) | Neutral |  |
|  | 548 (54.8) | 578 (39.9) | Very concerned/concerned |  |
